# Fruit odors enhance attractiveness of male *Drosophila melanogaster* flies during courtship

**DOI:** 10.1016/j.isci.2026.116768

**Published:** 2026-07-14

**Authors:** Julio Otárola-Jiménez, Markus Knaden

**Affiliations:** 1 Department of Evolutionary Neuroethology, Max-Planck Institute for Chemical Ecology, 07745 Jena, Germany; 2 Chemistry School, University of Costa Rica, San Pedro, San José 11501-2060, Costa Rica

**Keywords:** mating decision, vinegar fly, non-pheromonal attraction, assortative mating

## Abstract

Mate choice plays a critical role in reproductive success and species survival. In *Drosophila melanogaster*, female mating preferences are shaped by various factors, including male pheromones and experience. Here, we investigated whether exposure to distinct fruit-derived volatiles could influence male attractiveness. We found that fruits released unique volatile profiles and that males exposed to these substrates acquired detectable amounts of substrate-specific volatiles. When presented with a choice, females consistently preferred males previously exposed to apple volatiles over those exposed to pear or parsnip, with parsnip-exposed males being least attractive. Analysis of similarities revealed that body odor profiles of males reflected their respective substrates. Importantly, male exposure to different substrates was not associated with detectable differences in courtship latency or in pheromone levels. Our results suggest that environmentally acquired odors may affect female mate choice, pointing to a possible role for fruit-derived volatiles in modulating reproductive behavior in *Drosophila melanogaster*.

## Highlights

• Fruit-derived-odors act as natural perfumes for male vinegar flies
• Female mating decisions are modulated by these fruit-derived male odors
• Odors of some fruit enhance male attractiveness more than those of other fruit
• The observed change in male attractiveness is independent of male pheromones

## Graphical abstract

**Figure:**
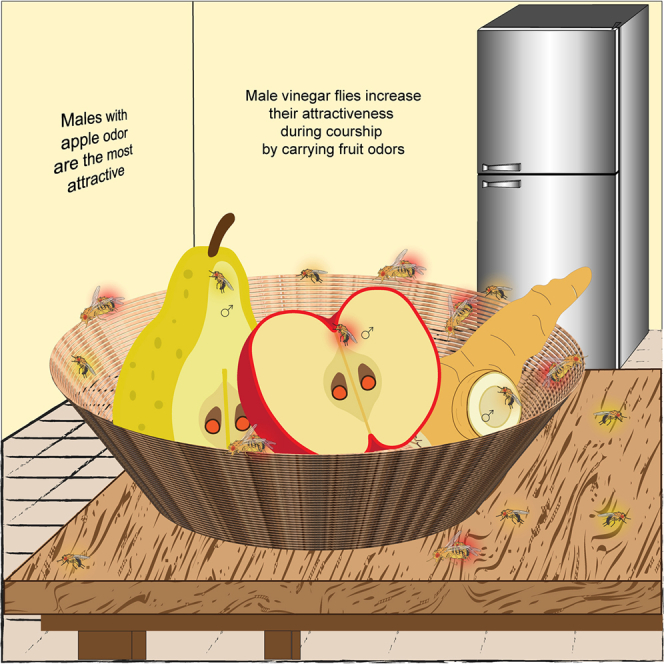

## Teaser

Biological sciences; Neuroscience; Behavioral neuroscience; Sensory neuroscience

## Introduction

Insects can communicate in different contexts, both between and within species, and through different modalities.^1^ Chemical signaling is one of the oldest and most common forms of insect communication, with pheromones governing communication within species and allomones between species.^2^ In drosophilid flies, males attempt to attract potential mates with different cues such as visual, acoustic, pheromonal, and other,^3^^,^^4^^,^^5^ while females typically have the final decision in choosing the most suitable partner. Numerous factors influence a female fly’s mating choice, including male experience,^6^ social context,^7^ and pheromones.^8^^,^^9^

In other species, such as *Anastrepha fraterculus* (a fruit fly of the family *Tephritidae*), exposure to fruit volatiles from guava, including (E)-β-ocimene, limonene or α-copaene, increases pheromone emission and, consequently, mating success.^10^ However, not all fruit odors produce the same effect. In this species, odors from fruit such as mango have no effect on mating success, whereas lemon odors can even reduce it.^11^

*Drosophila melanogaster* is often associated with various fruits, each releasing distinct volatile compounds that are detected through the fly’s olfactory system.^12^^,^^13^ It is well documented that chemical cues play a significant role in guiding female reproductive behavior.^14^ These chemical volatile cues can influence key behaviors such as oviposition.^15^ Recent studies have shown that fruits with high alcohol content promote the production of methylated pheromones—such as methyl laurate and methyl palmitate.^16^ We wondered whether apart from the effect of increasing the flies’ pheromones, fruit odors can themselves make a *Drosophila* male fly more attractive. Here, we investigate the role of fruit-derived odors in *Drosophila* mating behavior.

## Results

### Fruit-derived odors act as natural perfumes for male flies

**Figure 1:**
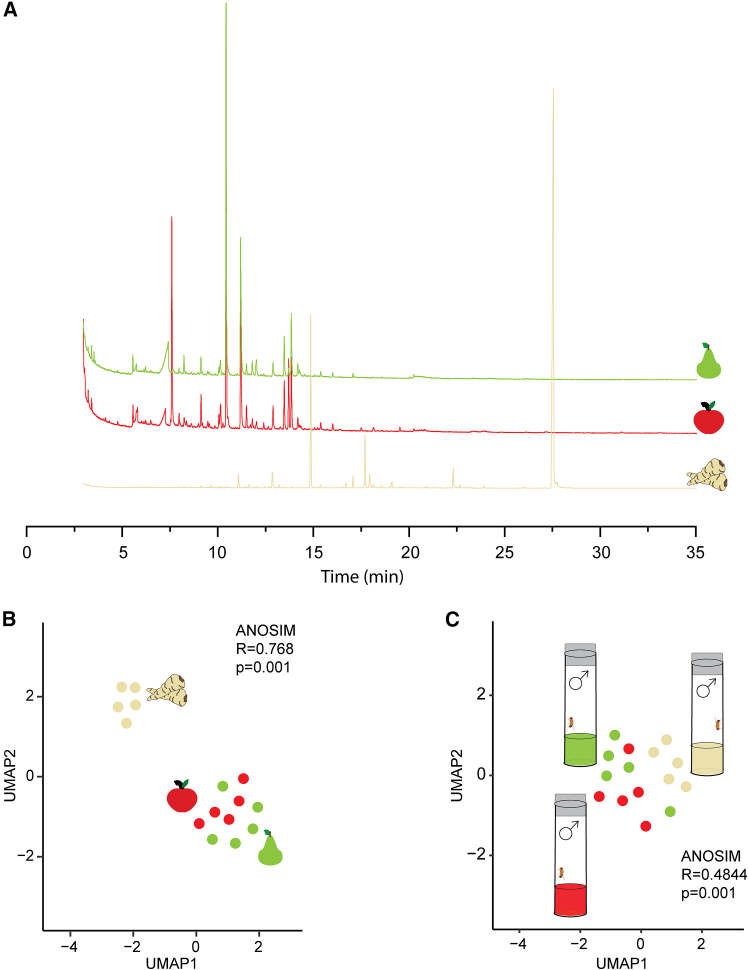
Volatile chemical profile of the substrates (A) Representative GC traces of volatiles collected from each substrate using dynamic headspace sampling followed by GC-MS analysis. Apple (red), pear (green), and parsnip (beige) each display distinct chemical profiles. (B) Uniform manifold approximation and projection (UMAP) clustering of the volatile profiles, with each point representing a single replicate (*n* = 5 per substrate). (C) UMAP clustering of the volatile profiles from males exposed to different substrates, with each point representing a single replicate that includes 50 males (*n* = 5 per substrate). Statistical differences in chemical composition between substrates were assessed using ANOSIM (analysis of similarity) with 999 permutations; *R* represents the test statistic, and *p* < 0.05 indicates a significant difference.

We first used pasteurized ripe but not fermented fruits to ensure that the substrates retained their natural chemical profiles without undergoing fermentation. We selected apple and pear, two well-known host substrates for *Drosophila melanogaster*, as well as parsnip, which is not a common natural host but has been shown to be attractive to gravid females when selecting an oviposition site.^17^ Each substrate emitted a distinct blend of volatile compounds (Figure 1A), with parsnip differing markedly from apple and pear (Figure 1B). Apple and pear volatiles were dominated by aldehydes and alcohols, while parsnip was rich in terpenes (Table S1).

Next, virgin males were placed individually in small vials containing one of three substrates for 24 h. Body odors from 50 treated males were then extracted with dichloromethane (DCM) and analyzed by gas chromatography (GC). Males exposed to different substrates showed subtle but reproducible differences in their odor profiles (Figure 1C, ANOSIM test, R∼0.5, *p* = 0.01), confirming that they retained volatiles from the substrate on which they were held.

### Fruit-derived odors adsorbed on male’s body modulate female mating decisions

Before assessing female mating preference for perfume-exposed males, we first tested female olfactory preference for fruit odors using a Y-maze (Figure S1A). Females showed no preference when apple was compared with parsnip (Figure S1B) or when pear was compared with parsnip (Figure S1D). However, when apple and pear were compared, females showed a significant olfactory preference for pear (Figure S1C).

**Figure 2:**
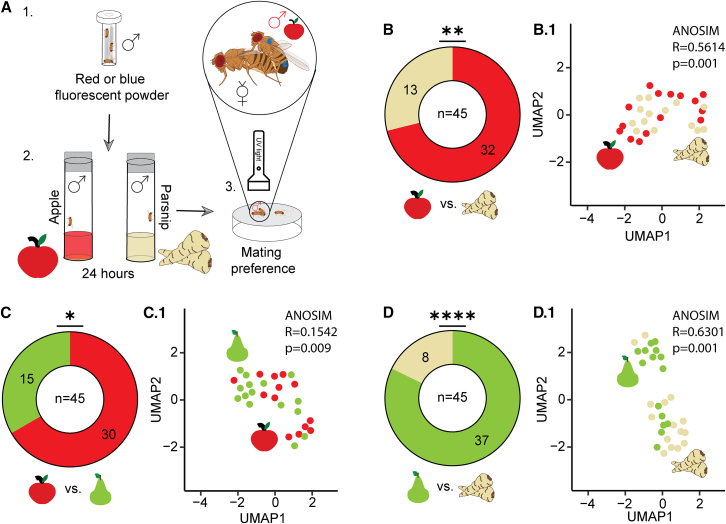
Fruit-derived odors influence female mate choice (A) Schematic of the experimental setup used to test female mating preference. Virgin males were exposed to one of three fruit substrates (apple, pear, or parsnip) for 24 h, then paired in mating arenas to compete for a single virgin female. (B–D) Female mating preferences when males from two different substrates were presented simultaneously: (B) apple (red) vs. parsnip (beige), (C) apple (red) vs. pear (green), and (D) pear (green) vs. parsnip (beige). Donut charts indicate the number of females that mated with males from each substrate. Numbers within the charts represent the count of successful matings per male type. Binomial tests were used to assess whether female choice deviated significantly from the null hypothesis of equal preference (expected ratio, 22.5/22.5). “∗” denotes significant differences with a *p* value ≤0.05, “∗∗” denotes significant differences with a *p* value ≤0.01, and “∗∗∗∗” denotes significant differences with a *p* value ≤0.0001. (B.1–D.1) UMAP clustering of the chemical profiles from male bodies after exposure to different substrates. Chemical data were collected using TDU-GC-MS, with each point representing an individual male (*n* = 13–15 per substrate). Statistical comparison of odor profiles was performed using ANOSIM with 999 permutations; R, average rank dissimilarity between groups, and *p* < 0.05 indicates a significant difference between groups.

We next examined whether these olfactory preferences translated into mating preferences. To do so, a single virgin female was offered a choice between two males that had been exposed to different substrates in a mating arena (Figure 2A), and her choice was recorded. The results showed that females preferred males exposed to apple over males exposed to pear or parsnip (Figures 2B and 2C), whereas males exposed to parsnip were consistently the least attractive (Figures 2B and 2D).

To determine whether males retained chemical signatures of their respective substrates, we analyzed single-male body volatiles using TDU-GC-MS. This analysis confirmed that males carried odor profiles reflecting the substrates to which they had been exposed, with myristicin from parsnip representing the most prominent peak. UMAP clustering of the chemical data showed separation between males from different substrates, with a strong and significant difference between apple/pear- and parsnip-exposed males (ANOSIM test, R > 0.5, *p* = 0.001; Figures 2B1 and 2D1), and a weaker but still significant difference between apple- and pear-exposed males (R < 0.5, *p* = 0.009; Figure 2C1). These findings mirror the differences observed when comparing the substrates directly (Figure 1B) and support the GC analysis of the fly body washes.

Because male mating success differed across treatments, we then asked whether this might also reflect diet-dependent differences in male courtship activity. Visual observations indicated that males were similarly active toward females across treatments. Consistent with this, we found no significant differences in courtship latency, defined as the time required for a male to initiate courtship, between males exposed to apple and parsnip (Figure S2), despite the marked nutritional differences between these substrates (Table S2). We also found no significant differences in mating latency, defined as the time to copulation (Figure S3), a measure associated with male attractiveness.^18^ Together, these results suggest that males from these treatments were similarly motivated to mate, making it less likely that broad differences in male activity explain the observed patterns of female choice.

This led us to test more directly whether olfactory perception contributes to these mating preferences. Fruit odors such as those from apple and pear are known to be detected by olfactory receptors (ORs).^13^ We therefore used *orco* mutant females in a mating assay comparing apple- and pear-exposed males (Figure S4). In contrast to wild-type females (Figure 2C), *orco* females showed no preference for males exposed to apple.

Together, these results support the idea that the chemical environment experienced by males, particularly exposure to volatiles from different substrates, can influence female mating decisions.

### Fruit-derived odors do not influence the pheromone profile

It was recently shown that fermented fruits with high methanol content enhance the production of three male pheromones: methyl dodecanoate (methyl laurate, ML), methyl tetradecanoate (methyl myristate, MM), and methyl hexadecanoate (methyl palmitate, MP).^16^ This prompted us to investigate whether male exposure to different substrates might influence their pheromonal chemical profiles.

**Figure 3:**
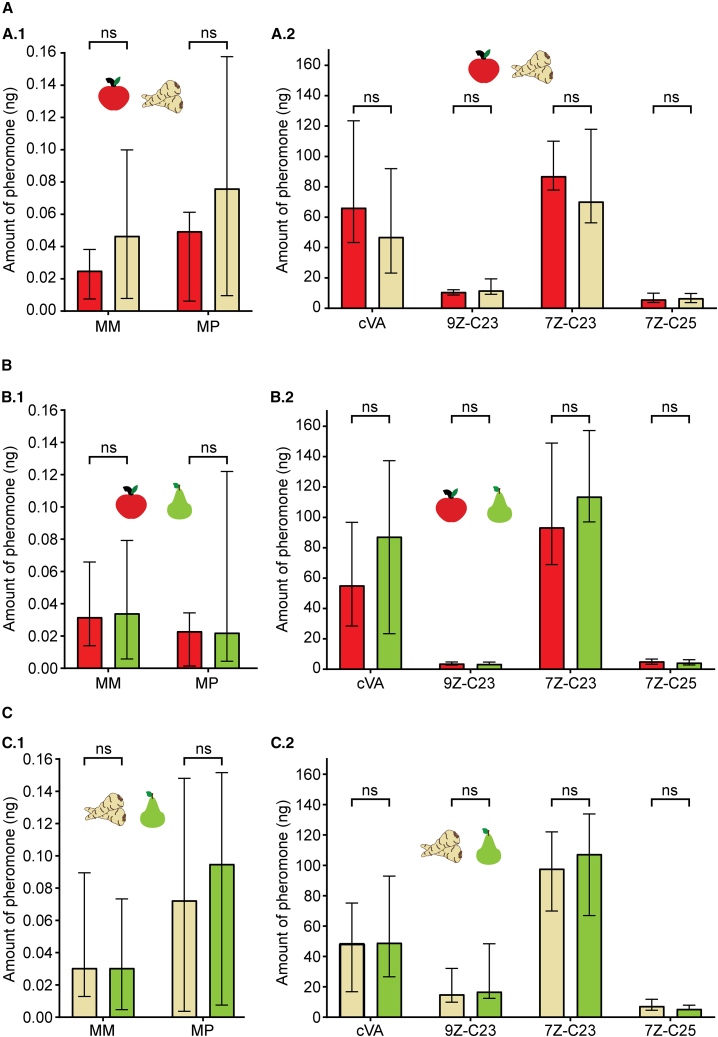
Male pheromone quantities were not affected by substrate exposure (A.1–C.1) The quantity in nanograms (ngs) of methyl myristate (MM) and methyl palmitate (MP) from males previously exposed to different substrates: (A.1) apple (red) vs. parsnip (beige), (B.1) apple (red) vs. pear (green), and (C.1) pear (green) vs. parsnip (beige). (A.2–C.2) The quantity in ngs of cVa, (Z)-tricos-9-ene (9Z-C23), (Z)-tricos-7-ene (7Z-C23), and (Z)-pentacos-7-ene (7Z-C25) from males previously exposed to different substrates: (A.2) apple (red) vs. parsnip (beige), (B.2) apple (red) vs. pear (green), and (C.2) pear (green) vs. parsnip (beige). Mann-Whitney tests revealed no significant differences in pheromone levels between groups. Bars indicate medians, and error bars represent 95% confidence intervals. Sample sizes per group ranged from *n* = 13–15. “ns” indicates no statistically significant difference detected.

To address this, we conducted thermal desorption sampling coupled with GC-mass spectrometry (TD-GC-MS) on male bodies from the different substrate treatments. Under our analytical conditions, we were able to detect and quantify MM and MP, but not ML (Figure 3), which is known to be produced in very low quantities.^9^^,^^16^

In addition to these methylated compounds, male flies are known to produce other pheromones, including the aggregation pheromone (7)-11-octadecenyl acetate (*cis*-vaccenyl acetate, cVA), (Z)-tricos-9-ene (9Z-C23), (Z)-tricos-7-ene (7Z-C23), and (Z)-pentacos-7-ene (7Z-C25),^14^^,^^19^^,^^20^ all of which we were also able to detect and quantify.

Our results show that cVA and 7Z-C23 were the most abundant pheromones across samples (Figures 3A2, 3B2, and 3C2), while the methylated compounds MM and MP were present in lower quantities (Figures 3A1, 3B1, and 3C1). Notably, we did not observe any significant differences in pheromone levels from males exposed to different diets. This is consistent with the idea that pasteurization may have removed potential sources of methanol and ethanol from the fruits, thereby limiting any diet-dependent effects on pheromone production under our experimental conditions.

Taken together, these results suggest that the differences in male attractiveness observed across diets are unlikely to be explained by variation in the measured male pheromones. Instead, they are more consistent with a role for other chemical cues, such as substrate-derived volatile compounds acquired on the male cuticle.

Overall, our findings indicate that female *Drosophila melanogaster* show substrate-dependent mating preferences, favoring males previously exposed to apple volatiles over those exposed to pear or parsnip. This pattern does not appear to be readily explained by differences in male activity or in the levels of the pheromones measured here. Rather, it suggests that environmentally acquired chemical cues may contribute to shaping female mate choice.

## Discussion

Female animals often control mating outcomes and typically favor male genotypes that confer to the offspring a higher fitness.^21^ In our study the male flies’ genetic background was held constant, and the substrates used during treatments were never used to rear any successive generation. Therefore, any shift in female preference had to arise just from the different environments the males were exposed to before mating.

Several proximate mechanisms for assortative mating in *Drosophila* have been described, including male-derived pheromones,^9^ female odor cues,^22^ ancestral diet effects,^23^^,^^24^ genotype-by-genotype compatibility,^25^ and courtship song differences.^26^ Our results suggest that environmentally acquired odors may represent an additional source of variation in male attractiveness. Males that spent only a few hours on different substrates carried distinct odor bouquets (Figure 1C), and these closely reflected the chemical differences among the substrates themselves (Figure 1B). Notably, these substrate-associated differences coincided with shifts in female mating decisions (Figure 2), suggesting that even relatively subtle changes in male odor profiles may influence mate choice.

This pattern is reminiscent of systems in which males incorporate environmental chemicals into sexual signaling. A well-known example is provided by orchid bees, whose males actively collect volatile compounds from their surroundings and store them in specialized hind-leg pouches, ultimately forming species-specific perfume blends used in courtship.^27^^,^^28^^,^^29^ Related observations have also been reported in tephritid flies such as *Bactrocera dorsalis* and *Anastrepha fraterculus*, where male exposure to fruit-derived odors can affect subsequent mating.^10^^,^^11^^,^^30^ Together, these examples raise the possibility that the use of environmentally derived chemical cues in sexual communication may be more widespread than is currently appreciated.

At the same time, an important distinction remains unresolved in our system: whether male *Drosophila* merely acquire these odors passively through contact with substrates, or whether they actively exploit particular substrates in ways that enhance their attractiveness. Addressing this question will be important for understanding the behavioral and ecological significance of this phenomenon. Future work should therefore test whether males preferentially visit or remain on certain fruits, and whether such behavior translates into consistent reproductive advantages under more natural conditions.

Keesey et al.^16^ showed that fermenting fruits have a high amount of methanol that can methylate endogenous *Drosophila* carboxylic acids and increase male attractiveness. In our study, however, we did not detect high amounts of methanol or ethanol, most likely because the food was pasteurized and hence did not ferment during the experiments. Consistent with this, total pheromone quantities (Figure 3) did not differ among males, despite being maintained for 24 h on chemically distinct, unfermented substrates.

Protein enrichment has been reported to increase precursor availability for pheromone production.^31^ Nevertheless, in our case, the parsnip diet, which had the highest content among the tested substrates, did not lead to increased pheromone quantities relative to apple or pear. Beyond diet, several other factors, including age, genotype, and microbial community, are known to influence the biosynthesis of another important pheromone, cVA.^32^ In our study, age was controlled (6–7 days old), and genotype was standardized by maintaining all flies on a common diet before treatments. Under these conditions, we did not detect any differences in cVA levels among males from the different substrate treatments.

Together, these observations suggest that the differences in mating preference we observed are unlikely to be explained solely by differences in overall pheromone abundance. Rather, they are consistent with the possibility that qualitative differences in cuticular volatiles acquired from the substrates contributed to variation in male attractiveness. More broadly, our findings raise the possibility that food-derived, fermentation-independent odor cues may influence mating outcomes and thus represent one factor contributing to sexual selection.^33^

To examine an alternative explanation, we next considered whether differences in the nutritional value of the substrates (Table S2) could have influenced male behavior. Apple and pear provide more energy than parsnip, largely because of their higher sugar content. If such nutritional differences had a strong effect on male activity, one might expect corresponding differences in courtship latency and mating latency. However, no such differences were detected when comparing males exposed to substrates with high energy (apple and pear) or low energy (parsnip) content (Figures S2 and S3). Nevertheless, we cannot exclude the possibility that diet affects more subtle components of the courtship repertoire.^34^^,^^35^^,^^36^

Further support for an odor-based interpretation comes from comparisons between substrates with similar nutritional profiles. Although apple and pear are similar in nutritional value (Table S2), wild-type females showed a significant preference for males exposed to apple (Figure 2C), whereas anosmic females (*orco* mutants) did not discriminate between males exposed to apple and pear (Figure S4). Taken together, these results support the interpretation that the preference shown by wild-type females for males exposed to apple may be influenced, at least in part, by substrate-derived odors carried by the males.

In our study, it remains unclear whether the adsorbed volatiles act as stand-alone aphrodisiacs or interact with canonical pheromones in a synergistic manner instead.^37^ However, female responses to odor substrates alone (Figure S1) did not fully mirror their preferences between males exposed to different substrates (Figure 2), a pattern that may be consistent with an interaction between substrate-derived odors and male pheromonal cues. This interpretation is broadly consistent with previous studies showing that food odors modulate several behaviors in *Drosophila*,^38^^,^^39^ such as enhancing female receptivity to courting males, although without directly affecting the copulation success.^37^^,^^40^ Thus, while females may be more attracted to males in the presence of food odors, this does not necessarily result in increased mating. In contrast, our results indicate that apple-derived odors associated with the male body were linked to female mating preference (Figure 2C). The discrepancy with previous studies may reflect differences in experimental design. In particular, earlier assays were performed in the presence of food, where feeding behavior itself may have influenced female responses, whereas our experiments were conducted without food present during mating. This design allowed us to focus more specifically on the effect of odor cues associated with prior substrate exposure. In addition, previous studies may have emphasized interactions between food odors and specific pheromones, such as cVA, whereas in our system substrate exposure did not measurably alter overall male pheromone abundance (Figure 3).

Our findings reveal a rapid, non-genetic route by which ecology can shape sexual selection. Merely spending a few hours on different, food substrates caused males to adsorb distinct bouquets of environmental volatiles. These qualitative odor cues—independent of any change in total pheromone output—were sufficient to shift female mating preferences. Whether these absorbed odors act alone or synergize with endogenous pheromones, our study underscores that “where a male has been” immediately before courtship can influence mate choice. At the same time, because our experiments were conducted under controlled laboratory conditions designed to isolate substrate-derived odors, further work will be required to determine how robust these effects are in more complex and ecologically realistic environments.

### Limitations of the study

This study focused on the effect of complete fruit-derived odor blends. However, future research is needed to determine whether the mating preference for males perfumed on apple is due to a specific chemical compound, a particular blend composition, or the concentration of certain volatiles—similar to what has been shown for oviposition preferences in citrus fruits.^15^ Additionally, it would be valuable to investigate if specific compounds from parsnip, such as terpenes, have an inherently unpleasant valence to virgin females, making males carrying these scents less attractive.^41^ Also, future studies should explore whether the adsorbed volatiles act as stand-alone aphrodisiacs or whether they act synergistically with males’ innate pheromones, as has been demonstrated for vinegar.^37^

An important limitation of this study is that the behavioral assays were conducted in a simplified laboratory setting using a two-choice design. While this approach allowed us to isolate the effect of substrate-derived odors, it does not capture the complexity of natural environments, where multiple males, diverse sensory cues, and microbial communities are present. Future studies should therefore investigate whether similar effects on male attractiveness are observed under more complex social conditions and in ecologically relevant contexts, including experiments with multiple competing males and in semi-natural or field settings.

Finally, a recent study showed that, in a competitive mating assay similar to ours, male-male rivalry mediated by agonistic wing flicks can play a more important role than female choice in determining mating outcomes.^42^ Importantly, that study also showed that such rivalry is influenced by olfactory information, including the amount of cVA. In our study, however, males were exposed to different substrate-derived odors while their main pheromone profiles remained similar. Future studies should therefore investigate whether agonistic wing flicks remain the decisive factor over female choice when males carry different substrate-derived odors.

## Resource availability

### Lead contact

Further information and requests for resources and reagents should be directed to and will be fulfilled by the lead contact, Markus Knaden (mknaden@ice.mpg.de).

### Materials availability

This study did not generate new unique reagents.

### Data and code availability

• The data described in this study have been deposited at Edmond-Research Data Repository of the Max Planck Society and is publicly available at DOI: https://doi.org/10.17617/3.KRTJYX as of the date of publication.
• All original code has been deposited at Edmond-Research Data Repository of the Max Planck Society and is publicly available at DOI: https://doi.org/10.17617/3.KRTJYX as of the date of publication.
• Any additional information required to reanalyze the data reported in this study is available from the lead contact upon request.

## Acknowledgments

We thank Daniel Veit for providing technical support in the device designs, Angela Lehmann for providing technical support in the GC analysis. We thank Ibrahim Alali for helping in the mating assays, Roland Spiess, and Regina Stieber-Rödiger for technical assistance. We also thank the 10.13039/501100001659Deutsche Forschungsgemeinschaft program FOR 5424 (MODOLFOR), the Office of International Affairs and External Cooperation at the University of Costa Rica (OAICE-UCR), the 10.13039/501100001655Deutscher Akademischer Austauschdienst (10.13039/501100001655DAAD), and the Max Planck Institute for Chemical Ecology for funding this work.

## Author contributions

Conceptualization, J.O.-J. and M.K.; methodology, J.O.-J. and M.K.; investigation, J.O.-J.; resources, M.K.; formal analysis, J.O.-J. and M.K.; writing – original draft, J.O.-J; writing – review and editing, M.K.; visualization, J.O.-J.; supervision, M.K. All authors read and approved the final manuscript.

## Declaration of interests

The authors declare no competing interests.

## STAR★Methods

### Key resources table

**Table:** 

REAGENT or RESOURCE	SOURCE	IDENTIFIER
**Experimental models: Organisms/strains**		

*D*. *melanogaster* (Dmel)	Hansson Lab (Canton S)	RRID:BDSC_1
*orco*^*KO*^ (*Or83b*^*KO*^)	Bloomington *Drosophila* Stock Center BL23129 (Line 642 Hansson Lab)	RRID:BDSC_23129

**Software and algorithms**		

R 4.4.3	https://www.r-project.org/	RRID:SCR_001905
RStudio 2025.09.0	https://www.r-project.org/	RRID:SCR_000432
GraphPad Prism 10.2.3	https://www.graphpad.com/	RRID:SCR_002798
Adobe Illustrator 29.8.7	https://www.adobe.com/	RRID:SCR_010279

### Experimental model and study participant details

#### *Drosophila melanogaster*

This study used wild-type flies that were acquired from the National *Drosophila* Species Stock Center (NDSSC; http://blogs.cornell.edu/drosophila/): *D*. *melanogaster* (Canton S, Hansson’s Lab). Also *orco*^*KO*^ (*Or83b*^*KO*^) flies were used that were acquired from the Bloomington *Drosophila* Stock Center (BDSC;BL23129; https://bdsc.indiana.edu/): *orco-*mutant (Line 642, Hansson’s Lab). All the flies were reared on yeast-cornmeal-agar medium (standard food∗), kept at 25°C with 70% humidity. A 12 h:12 h light:dark cycle was established. Flies were collected and sexed during the first 3 h after eclosion, using ice as an anesthetic. Groups of 5 female and 5 male flies were kept separately in small vials with standard food until the start of the experiments. Flies were treated under the same conditions for all experiments.

∗For 1 L of the standard food, the following ingredients were used: beet syrup (118 g), brewer’s yeast (11 g), agar powder (4.1 g), hot water (540 mL), corn grits (95 g), propionic acid (2.4 mL), nipagin 30% (3.3 mL), and cold water (378 mL).

### Method details

#### Food preparation

Purchasable purees of apple, parsnip, and pear (Hipp, Germany) were used as substrates (See Table S2 for nutritional information). For each experiment, a freshly prepared substrate was used, in which the puree was combined with agarose to reach similar consistency. To prepare 100 g of substrate, 0.4 g of agarose (Agar-Agar, Kobe I Art.Nr 5210.2 Carl Roth GmbH) and 27 mL of distilled water were boiled. When this mixture reached ∼60°C, 80 g of the puree was added and mixed. Then, 4.5 g of this mixture were transferred into narrow *Drosophila* tubes (28.5 × 95 mm, Dominique Dutscher) or 35 × 10 mm Petri dishes (Ref. No. 734–2314, VMR).

#### Competitive mating assays

We followed a protocol similar to that described by Khallaf et al.^43^ Briefly, 6–7-day-old males were marked 24 h before the experiment using UV-fluorescent powders of different colors (red: UVXPBR, blue: UVXPBB; Maxmax.com: https://maxmax.com/shopper/category/9483-uv-powders). After marking, individual males were placed in narrow *Drosophila* tubes (28.5 × 95 mm, Dominique Dutscher) containing 4.5 g of a given substrate and kept there for 24 h. To avoid potential bias associated with fluorescent dyes, we swapped the fluorescent powders assigned to each substrate every ten replicates.

The following morning, between 8:00 and 10:00 a.m., one male from each of two different substrate treatments and one virgin female (wild type or *orco-*mutant) were introduced into a mating chamber (1 cm diameter × 0.5 cm depth) covered with a plastic slide. Mating assays were observed manually for 1 h and copulation success was determined under UV light by identifying the color of the successful male (Figure 2A). Courtship latency (time to start the courtship) and mating latency (time to start the mating) was measured using a digital stopwatch (related to Figures S2 and S3).

#### Y-maze assays

In order to test the innate olfactory preference of virgin females (wild type) for the headspace of a given substrate, we performed a Y-maze assay (Figure S1). We followed a protocol similar to that described by Otárola-Jiménez et al. (2024).^17^ Briefly, two 300 mL/min airflows were used and bubbled through a deionized water reservoir to create a moist airflow. One moist airflow was connected to a bottle with 40 g of one substrate. Another airflow was connected to a bottle with 40 g of another substrate. Afterward, both airflows passed through plastic traps. Finally, the airflows reached both arms of the Y-maze. The odor preference of individual virgin female flies was measured based on which trap the flies entered within 5 min. For each replicate, a single fly was put into the Y maze with an aspirator and allowed to acclimatize for 30 s. Experiments were conducted between ZT5 and ZT9. Only flies that reached the odor-trap within 5 min of test were used for the analysis. After five tested flies the odorants were switched between trials to control for a potential side bias.

#### Collection of substrate`s odors (dynamic headspace)

We followed a protocol similar to that described by Depetris-Chauvin et al.^44^ and Bisch-Knaden et al.^45^ Briefly, a 35 × 10 mm Petri dish (Ref. No. 734–2314, VMR) with 4.5 g of a given substrate was enclosed in polyethylene terephthalate bags (Toppits, Germany). Headspaces were collected for 24 h using a push-pull system with a Super-Q filter (50 mg, www.volatilecollectiontrap.com) and a charcoal filter. Then, the Super-Q filter was eluted with 4 × 100 μL dichloromethane.

Headspace samples were analyzed by GC-MS (6890 N GC system, 5975 B MSD, Agilent Technologies, https://www.agilent.com) equipped with a semi-polar column (HP5-Ultra Inert, 5% phenyl-polymethylsiloxane, 30 m long, 0.25 mm inner diameter, 0.25 μm film thickness; Agilent 19091 S-433UI) with helium as carrier gas. The inlet temperature was set to 240°C. The temperature of the GC oven was held at 40°C for 3 min, and then increased by 5°C per min to 260°C. This final temperature was held for 5 min. The MS transferline was held at 280°C, the MS source at 230°C, and the MS quad at 150°C. Mass spectra were taken in electro ionization mode (70 eV) in the range from *m/z* 29 to 350. GC-MS data were processed with the MDS-ChemStation Enhanced Data Analysis software (Agilent). Identification of volatile compounds was performed by comparing the mass spectra with those from the database (NIST 23), and retention index (RI) values of the detected compounds were determined by comparison of the retention times with those for a series *n*-alkanes saturated standard (C7-C40, Sigma Aldrich, 49452-U), which was analyzed in a separate analysis under the same conditions (related to Figures 1A and 1B).

#### Collection of body`s odors (body wash)

Flies aged 5–6 days were individually placed in narrow *Drosophila* tubes (28.5 × 95 mm, Dominique Dutscher) containing 4.5 g of a given substrate and kept there for 24 h. After exposure, 50 flies from the same treatment were pooled into a 1.5 mL vial (ref. 702283, Macherey-Nagel GmbH & Co.) and frozen for 20 min. Subsequently, 400 μL of dichloromethane were added, and the vial was vortexed (Vortex-Genie 2) for 3 min to extract body odors. The resulting extract was filtered using a disposable syringe filter (Chromafil PTFE-20/3, 0.2 μm pore size, ref. 729014, Macherey-Nagel GmbH & Co.) and stored at −20°C until analysis.

Each sample was analyzed by gas chromatography coupled with mass spectrometry (6890 N GC system, 5975 B MSD, Agilent Technologies (https://www.agilent.com), using the same conditions as those described for substrate odor collection (related to Figure 1C).

#### Analysis of pheromone compounds (TDU-GC-MS)

We followed a protocol similar to that described by Dweck, Ebrahim, Thoma et al.^9^ for a TD-GC-MS analysis. Briefly, individual males that were previously on a given substrate for 24 h were placed in standard microbial in thermal desorption tubes and transferred using a GERSTEL MPS 2 XL multipurpose sampler into a GERSTEL thermal desorption unit (www.gerstel.de). 2 μL of 2.36 ng/μL 1-bromodecane as internal standard was added to each fly before the desorption to quantify the pheromones of interest. After desorption at 200°C for 5 min free of solvent, the analytes were trapped in the liner of a GERSTEL CIS 4 Cooled injection system at −50°C, using liquid nitrogen for cooling. The components were transferred to the GC column by heating the programmable temperature vaporizer injector at 12°C per second up to 240°C and then held for 5 min. The GC-MS device (Agilent GC 7890 A fitted with an MS 5975C inert XL MSD unit; www.agilent.com) was equipped with an HP5-MS UI column (19091 S-433UI; Agilent Technologies) and operated as follows. The temperature of the gas chromatograph oven was held at 40°C for 3 min and then increased by 5°C per min to 260°C. This final temperature was held for 1 min and then increased by 10°C per min to 300°C and held for 5 min. The MS transfer line was held at 280°C, the MS source at 230°C, and the MS quad at 150°C. Mass spectra were taken in electro ionization mode (70 eV) in the range from *m/z* 33 to 350. GC-MS data were processed with the MDS-ChemStation Enhanced Data Analysis software (Agilent). Identification of volatile compounds was performed by comparing the mass spectra with those from the database (NIST 23), and retention index (RI) values of the detected compounds were determined by comparison of the retention times with those for a series *n*-alkanes saturated standard (C7-C40, Sigma Aldrich, 49452-U), which was analyzed in a separate analysis under the same conditions (related to Figure 2).

Commercial standard of methyl myristate (MM, 99% pure, Fluka, item# 15560634, www.fishersci.de), methyl palmitate (MP, 98.5% pure, SigmaAldrich, item# 76159, www.sigmaaldrich.com), *cis-*vaccenyl acetate (cVa, 99% pure, Pherobank, item# 10421, www.pherobank.com), (Z)-tricos-9-ene (9Z-C23, 90% pure, Cayman Supports Science, item# 13236, www.caymanchem.com), (Z)-tricos-7-ene (7Z-C23, 95% pure, Cayman Supports Science, item# 9000313, www.caymanchem.com), and (Z)-pentacos-7-ene (7Z-C25, 98% pure, Cayman Supports Science, item# 9000530, www.caymanchem.com) were used to confirm the retention time of the peaks of interest (related to Figure 3).

### Quantification and statistical analysis

Statistical analysis tests, sample sizes, and corrections for multiple comparisons are given in the text and figure legends. Statistical tests and data visualization were performed with R (R version 4.4.1 (2024-06-14 ucrt)), GraphPad Prism (version 10.2.3 (2024-04-21), and OriginPro 2023 (10.0.0.154). Figures were then processed with Adobe Illustrator.

To perform the Uniform Manifold Approximation and Projection (UMAP) analysis (Figures 1, 2, and 3) with the GC-MS information, first a *XCMS* method was used, following similar steps as published by Moreira-Soto et al.^46^

• The raw GC/MS data were converted to AIA format using MSD ChemStation (Agilent Technologies).
• These converted files were imported into R (version 4.4.1) where the XCMS package was employed for peak detection and retention time alignment.
• For peak detection in XCMS, the *centWave* algorithm was applied, setting a mass accuracy of 20 ppm, peak width between 3 and 50 seconds, and a signal-to-noise threshold of 20.
• Then, for Figure 1, a retention time filterering was applied to include only peaks eluting between 150 and 3500 seconds. While for Figure 2 a filter between 150 and 1800 seconds was used.
• The resulting dataset was grouped using a density-based algorithm with parameters set to a retention time bandwidth (*bw*) of 10, a minimum fraction of 0.1, a minimum of one sample per group, and a mass accuracy of 0.1.
• Then, retention time alignment across samples was performed using the *rector()* function with the method *obiwarp* and a profile step of 0.5.
• A second round of grouping was conducted on the aligned dataset using the same density method but with a narrower retention time bandwidth (*bw* = *5*).
• To aid compound annotation, peak correlation grouping was conducted using *CAMERA* package. The *xsAnnotate()* function was used to initialize the annotation object, and peak groups were refined using full width at half maximum grouping (*groupFWHM()*) with a performance width threshold of 0.6.
• The processed data were converted to a data frame using *getPeaklist()* and reformatted for further analysis (UMAP).
• Peak intensities and features with distinct *m/z* (mass-to-charge ratios) were normalized by the sum of all features per sample using a custom function (*calculate*.*sums()*), assuming a total of 30 samples. The resulting peak intensity summaries were exported as CSV file (*sums_Prueba*.*csv*). (See supplementary material for script).

To visualize high-dimensional differences among odor profiles, a Uniform Manifold Approximation and Projection (UMAP) analysis was performed using the *uwot* package. The group column was converted to a factor, and all feature values were standardized using *Z* score scaling. UMAP was conducted with 15 nearest neighbors, two output dimensions, and the Bray-Curtis dissimilarity metric. A minimum distance of 0.01 was set to preserve local structure. The resulting coordinates were plotted using *ggplot2*, and data points were colored by group.

To statistically evaluate group separation, Analysis of Similarities (ANOSIM) was performed using the *vegan* package. A Bray-Curtis dissimilarity matrix was calculated from the feature matrix, and group differences were tested using 999 permutations (see Edmond database (https://doi.org/10.17617/3.KRTJYX) for script).
