# Supplementary material for: Fruit odors enhance attractiveness of male Drosophila melanogaster flies during courtship
Source: iScience. 2026 Jul 14;29(8):116768. doi: 10.1016/j.isci.2026.116768 (PMC13382592; doi:10.1016/j.isci.2026.116768)
Supplement: Document S1. Figures S1–S4, Tables S1, and S2 [file mmc1.pdf]

iScience, Volume *29*

Supplemental information

Fruit odors enhance attractiveness of male *Drosophila melanogaster* flies during courtship

Julio Otárola-Jiménez and Markus Knaden

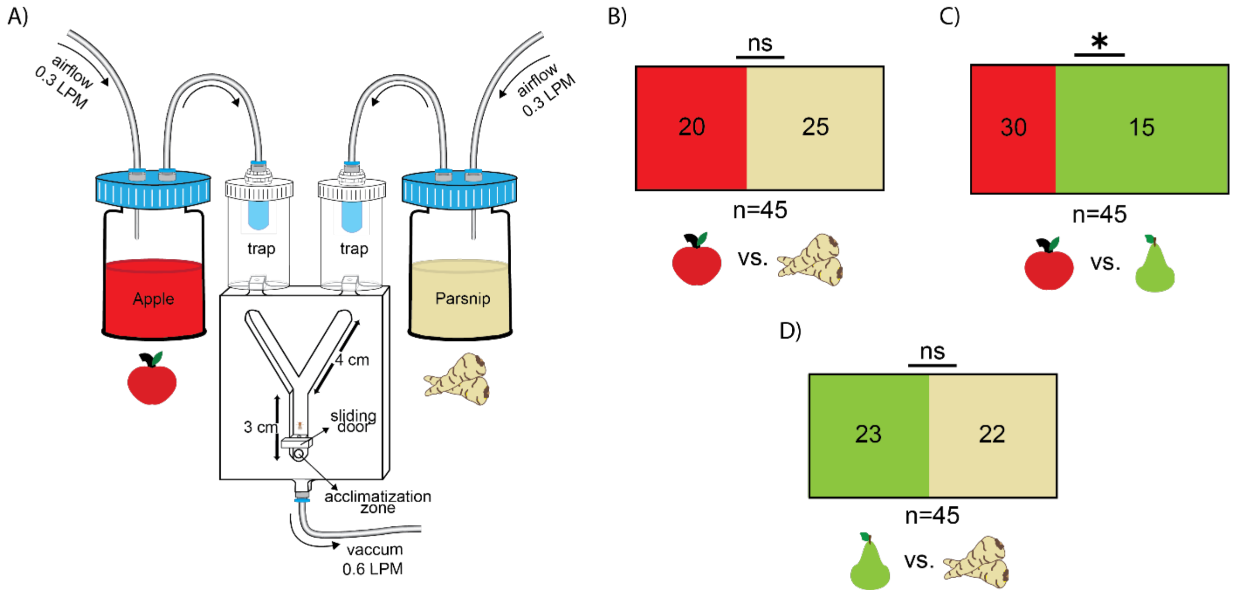

Figure S1: Innate fruit-odor preference of virgin female flies, related to figure 1. (A) Schematic of the Y-maze used to test olfactory attraction of individual virgin females before mating. Flies were released into the acclimatization zone of the entrance arm; after 30 s, the opening of a wind permeable sliding door enabled flies to reach the bifurcation of the Y-maze, where they could choose between the headspace from both substrates. Entering one of the traps was regarded as decision. Each fly had 5 min to decide. Adapted from Otárola-Jiménez, *et al.*, 2024[S1]. (B) Olfactory preference of naïve virgin flies between apple (red) and parsnip (beige). (C) Olfactory preference of naïve virgin flies between apple (red) and pear (green). (D) Olfactory preference of naïve virgin flies between pear (green) and parsnip (beige). (B-C) n below each bar graph, number of tested flies; number in each section of bar graph, number of flies that chose each odor. Binomial tests (two-tailed) were used to assess whether female choice deviated significantly from the null hypothesis of equal preference (expected ratio: 22.5/22.5). "*" denotes significant differences with a p-value ≤ 0.05, and "ns" denotes non-significant differences.

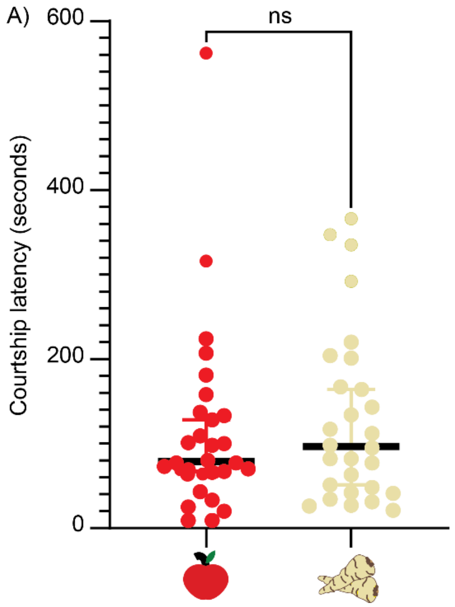

Figure S2: Male exposure to different substrates did not affect courtship, related to Figure 2B. Shows courtship latency of males when females were presented with two males previously exposed to different substrates, apple (red) vs. parsnip (beige). No significant differences were found in any comparison (Wilcoxon matched-pairs signed rank test, $p > 0.05$). Black lines indicate medians, and error bars represent 95% confidence intervals. Each group consisted of $n = 27$ replicates. "ns" denotes non-significant differences.

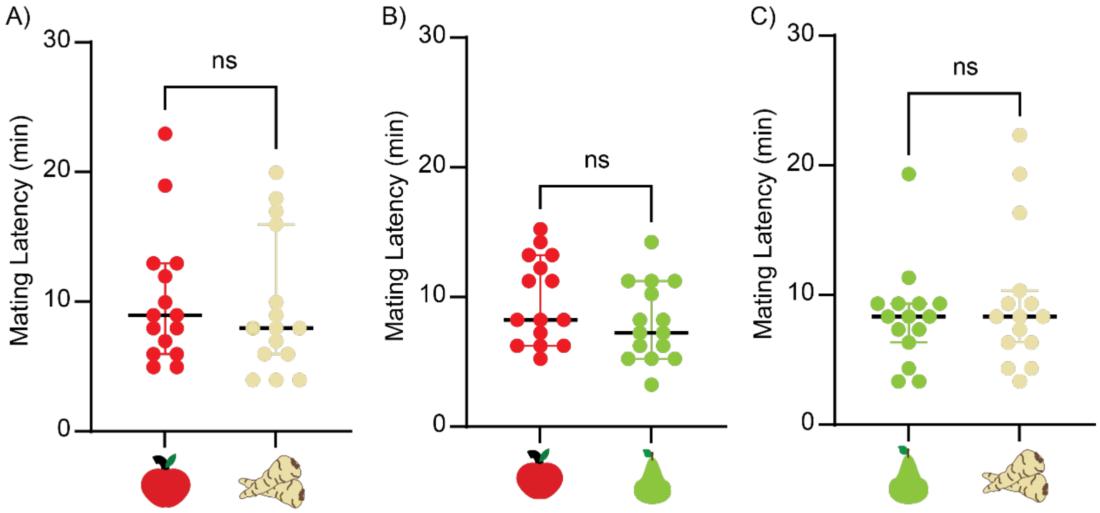

Figure S3: Mating latency is not affected by diet differences, related to Figure 2. Females were presented with two males previously exposed to different substrates: (A) Apple (red) vs. parsnip (beige), (B) Apple (red) vs. pear (green), and (C) Pear (green) vs. parsnip (beige). No significant differences were found in any comparison (Wilcoxon rank-sum test, $p > 0.05$). Black lines indicate medians, and error bars represent 95% confidence intervals. Each group consisted of $n = 15$ replicates. "ns" denotes non-significant differences.

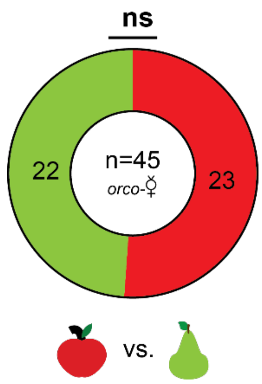

Figure S4: *Orco*-female does not differentiate males' perfume with apple and pear during courtship, related to Figure 2C. Donut chart indicate the number of females that mated with males from apple (red) or pear (green). Numbers within the chart represent the count of successful matings per male type. Binomial tests were used to assess whether female choice deviated significantly from the null hypothesis of equal preference (expected ratio: 22.5/22.5). "ns" denotes non-significant differences.

Table S1: List of volatile compounds in the dynamic headspace of substrates, related to Figure 1A. Chemical compounds are listed by retention time (RT) and the type of substrates. Compounds were identified by MS and KI (Kovats index) using an alkane standard (C7-C30) under the same conditions.

		**Apple**		
#Peak	*Retention Time (min)*	*Compound*	*identification by*	*% Area*
1	3.355	pentanal	MS and KI	0.697
2	3.481	2-Ethoxyethanol	MS and KI	0.644
3	5.523	hexanal	MS and KI	0.339
4	9.082	?	-	1.445
5	10.017	Octane, 3-methyl-6-methylene-	MS and KI	0.705
6	10.101	1-Isobutyl-3-methylcyclopentane	MS and KI	1.862
7	10.394	(E)-2-heptenal	MS and KI	43.088
8	11.155	1-octen-3-one	MS and KI	20.78
9	11.463	6-Methyl-5-heptene-2-one	MS and KI	0.931
10	11.769	(E,E)-2,4-Heptadienal	MS and KI	2.251
11	12.849	2-Ethyl-1-hexanol	MS and KI	1.807
12	13.436	5-methyl-1-heptanol	MS and KI	7.071
13	13.809	(E)-2-Octenal	MS and KI	7.726
14	14.142	(2Z)-2-Octen-1-ol	MS and KI	1.311
15	15.339	Nonanal	MS and KI	1.818
		**Pear**		
#Peak	*Retention Time (min)*	*Compound*	*identification by*	*% Area*
1	7.199	Butanoic acid, 2-methyl-	MS and KI	11.91
2	9.081	?	-	2.708
3	10.101	1-Isobutyl-3-methylcyclopentane	MS and KI	2.84
4	10.395	(E)-2-Heptenal	MS and KI	44.087
5	11.16	1-Octen-3-one	MS and KI	17.764
6	11.463	6-Methyl-5-heptene-2-one	MS and KI	2.441
7	11.987	octanal	MS and KI	1.941
8	12.85	2-Ethyl-1-hexanol	MS and KI	3.138
9	13.442	5-Methyl-1-heptanol	MS and KI	3.566
10	13.812	(E)-2-Octenal	MS and KI	5.622
11	15.338	Nonanal	MS and KI	1.99
		**Parsnip**		
#Peak	*Retention Time (min)*	*Compound*	*identification by*	*% Area*
1	9.083	?	-	0.157
2	9.612	alfa-pinene	MS and KI	0.153
3	10.018	2-Ethyl-5-methyl-1-heptene	MS and KI	0.083
4	10.105	1-Isobutyl-3-methylcyclopentane	MS and KI	0.145
5	11.047	beta-pinene	MS and KI	0.978
6	11.382	4-Methyl-3-cyclohexen-1-one	MS and KI	0.11
7	11.588	beta-myrcene	MS and KI	0.195
8	11.995	alfa-Phellandrene	MS and KI	0.13
9	12.417	?	-	0.17
10	12.684	p-cymene	MS and KI	0.116
11	12.818	d-limonene	MS and KI	1.165
12	13.163	(3*Z*)-3,7-dimethylocta-1,3,6-triene	MS and KI	0.233
13	13.834	γ-Terpinene	MS and KI	0.156
14	14.82	Terpinolene	MS and KI	15.57
15	15.335	?	-	0.138
16	16.47	?	-	0.242
17	16.664	?	-	0.292
18	17.654	cis-p-mentha-1(7),8-dien-2-ol	MS and KI	5.635
19	17.905	p-Cymen-8-ol	MS and KI	1.065
20	18.083	(-)-α-Terpineol	MS and KI	0.343
21	18.971	p-cumenol	MS and KI	0.179
22	19.061	?	-	1.088
23	22.274	?	-	1.694
24	23.879	(6E)-6-Methyl-5-(1-methylethylidene)-6,8-nonadien-2-one	MS and KI	0.172
25	27.482	Myristicin	MS and KI	69.539

Table S2: Nutritional information of Hipp purees, related to Figure 2. Nutritional values reported by the commercial company (https://www.hipp.de/). These products contain "no artificial flavors, no preservatives, and no artificial colors", according to the manufacturer.

**Apple**: 100 % Bio-apple	
Content average	*per 100 g*
Energy kJ/kcal	211/50
Fat	0.1 g
-of which, saturates	0.0 g
Carbohydrate	11.2 g
-of which, sugar	10.7 g
Protein	0.2 g
Salt	< 0.05 g
Sodium	< 0.02 g
**Parsnip**: 70 % Bio-parsnip, 30% water	
Content average	*per 100 g*
Energy kJ/kcal	126/30
Fat	0.3 g
-of which, saturates	0.0 g
Carbohydrate	5.2 g
-of which, sugar	2.7 g
Protein	0.6 g
Salt	< 0.05 g
Sodium	< 0.02 g
**Parsnip**: 100 % Bio-Williams-Christ pear	
Content average	*per 100 g*
Energy kJ/kcal	231/55
Fat	0.3 g
-of which, saturates	0.0 g
Carbohydrate	11.0 g
-of which, sugar	9.1 g
Protein	0.5 g
Salt	< 0.05 g
Sodium	< 0.02 g

Supplemental reference

[S1]. Otárola-Jiménez, J., Nataraj, N., Bisch-Knaden, S., Hansson, B.S., and Knaden, M. (2024). Oviposition experience affects oviposition preference in Drosophila melanogaster. iScience *27*, 110472. https://doi.org/10.1016/j.isci.2024.110472.
